# Preventive Effect of Preoperative Vitamin D Supplementation on Postoperative Atrial Fibrillation

**DOI:** 10.21470/1678-9741-2018-0014

**Published:** 2018

**Authors:** Levent Cerit, Barçın Özcem, Zeynep Cerit, Hamza Duygu

**Affiliations:** 1 Department of Cardiology, Near East University, Nicosia, Cyprus.; 2 Department of Cardiovascular Surgery, Near East University, Nicosia, Cyprus.; 3 Department of Pediatric Cardiology, Near East University, Nicosia, Cyprus.

**Keywords:** Atrial Fibrillation, Coronary Artery Bypass, Vitamin D

## Abstract

**Objective:**

To assess the relationship between preoperative vitamin D (vitD) supplementation and the development of postoperative atrial fibrillation (POAF).

**Methods:**

The study group consisted of 328 consecutive patients. The ınfluence of preoperative vitD supplementation on POAF was reviewed in 136 patients who underwent coronary artery bypass graft surgery with vitD insufficiency (n=80) and vitD deficiency (n=56). Patients were assigned to receive either oral vitD (50.000 U) (treatment group, n=68) or not (control group, n=68) 48 hours before surgery. Patients were followed up during hospitalisation process with respect to POAF.

**Results:**

There was no significant difference between treatment and control groups with regards to age, gender, diabetes mellitus, smoking history, chronic obstructive pulmonary disease, left atrial diameter, and biochemical parameters. Also, there was no significant difference between these groups with regards to mean vitD level on both insufficiency and deficiency patients (24.6±3.7 *vs*. 24.9±3.9 ng/ml *P*=0.837, 11.4±4.9 *vs*. 10.9±5.2 ng/ml *P*=0.681, respectively). Although the occurrence of POAF was not significantly different among treatment and control groups in patients with vitD insufficiency (31% *vs*. 33% *P*=0.538), there was a significant difference between the two groups regarding to POAF in patients with vitD deficiency (18% *vs*. 29% *P*=0.02).

**Conclusion:**

Although preoperative vitD supplementation was not found to be associated with prevention of POAF in patients with vitD insufficiency, it was found to be strongly associated with prevention of POAF in those with vitD deficiency.

**Table:** 

Abbreviations, acronyms & symbols				
ACE-i/ARB	= Angiotensin-converting enzyme inhibitor/		HT	= Hypertension
	angiotensin receptor blockers		IL	= Interleukin
AF	= Atrial fibrillation		MPV	= Mean platelet volume
CABG	= Coronary artery bypass graft		25-(OH)	= 25-hydroxy (OH)
COPD	= Chronic obstructive pulmonary disease		POAF	= Postoperative atrial fibrillation
DM	= Diabetes mellitus		RAAS	= Renin-angiotensin-aldosterone system
ECG	= Electrocardiogram		RDW	= Red blood cell distribution width
EF	= Ejection fraction		SD	= Standard deviation
HF	= Heart failure		TIA	= Transient ischemic attack
HL	= Hyperlipidemia		vitD	= Vitamin D

## INTRODUCTION

Atrial fibrillation (AF) is the most common arrhythmia occurring after coronary artery bypass graft (CABG) surgery and it is seen in approximately 15%-60% of these patients. Postoperative AF (POAF) is associated with increased morbidity, mortality, and longer hospital stay; it is also associated with a two-to-three-fold increase in postoperative stroke. Ageing, obesity, hypertension (HT), prior AF, and heart failure (HF) are found strongly associated with higher risk of POAF^[1]^.

Vitamin D (vitD) has various cardiovascular pleiotropic effects by activating its nuclear receptor in cardiomyocytes and vascular endothelial cells and by regulating the renin-angiotensin-aldosterone system (RAAS), adiposity, and energy expenditure. There is inconsistent data about the association between vitD and POAF. POAF incidence was significantly higher in patients with vitD deficiency or insufficiency than in patients with normal vitD level in previous studies^[2-6]^. In our previous study, although there was a significant negative correlation between vitD and left atrial diameter, the vitD level was not an independent predictor of POAF^[7]^.

To the best of our knowledge, the relationship between preoperative vitD supplementation in patients with vitD deficiency/insufficiency and POAF has not been studied before. Therefore, we assessed the preventive effect of vitD supplementation on POAF in patients who underwent CABG surgery with vitD deficiency/insufficiency.

## METHODS

Our study was a randomised, blinded clinical trial. The study group consisted of 328 consecutive patients who underwent on-pump CABG surgery. Prevalence of vitD insufficiency and deficiency found were 24.3% (n=80) and 17% (n=56) among the study population, respectively. Patients were assigned to receive either oral vitD (50.000 IU) (treatment group; n=40 insufficiency patients, n=28 deficiency patients) or not 48 hours before CABG surgery. Patients were followed up during hospitalisation process with respect to new-onset AF. All investigators were blinded to treatment assignment. VitD supplementation was initiated in control group while discharging. The patients' data were analysed for AF in the postoperative period until discharge. The study was approved by the local ethics committee.

### Definition of POAF

The study's main outcome was the occurrence of POAF from surgery until hospital discharge. Continuous electrocardiogram (ECG) monitoring was performed up to 48 hours after the operation to detect new-onset AF. Subsequently, telemetric ECG device was placed on the patients until the fifth postoperative day. The presence of ECG-documented AF for at least 5 min was recorded and analysed as POAF without symptoms. All symptomatic episodes detected by the patients or clinical residents were confirmed by a 12-lead ECG, and at least 1 min was recorded and analysed as POAF. Patients who developed AF in the postoperative period until discharge were included in the POAF group.

Patients who developed POAF were treated with intravenous beta-blocker as a first-line rate control therapy. Intravenous amiodarone was initiated as a first-line rhythm control therapy. If the heart rate was not tolerated despite medical therapy (amiodarone, beta-blocker), electrical cardioversion was performed if POAF had been occurred for <48 hours. If POAF had been occurred for ≥48 hours, left atrial clot was ruled out with transesophageal echocardiography before electrical cardioversion. Anticoagulation therapy was initiated in patients with POAF and continued for at least three months. The decision to continue oral anticoagulation after three months was made according to the CHA2DS2-VASC score [congestive HF, HT, age ≥75 years (2 points), diabetes mellitus (DM), previous stroke/transient ischemic attack (TIA) (2 points), vascular disease, age 65-74 years, and gender (female)].

Patient's data including age, gender, history of HT, hyperlipidemia (HL), chronic kidney disease, DM, HF, chronic obstructive pulmonary disease (COPD), congenital heart disease, valvular heart disease, liver disease, stroke, thyroid disease, preoperative drug use [beta-blockers, diuretics, aspirin, angiotensin-converting enzyme inhibitor/angiotensin receptor blockers (ACE-i/ARB), and statins], echocardiographic variables such as ejection fraction (EF) and left atrial diameter, and presence of valvular disease were retrieved from the medical charts and included in the analysis.

### Echocardiographic Examination

All patients underwent transthoracic echocardiography using Vivid S5 (GE healthcare) echocardiography device and Mass S5 probe (2-4 MHz). Standard two-dimensional and colour flow Doppler evaluation were acquired according to the American Society of Echocardiography and European Society of Echocardiography guidelines^[8]^. EF was measured according to the Simpson's method. Left atrial diameter was measured in parasternal long axis view using two-dimensional echocardiography at the left ventricular end-systole.

The study's exclusion criteria were: patients with paroxysmal or persistent AF, being on antiarrhythmic medications, patients who underwent pharmacological or electrical cardioversion before CABG surgery due to reasons other than AF, and patients who underwent other cardiac procedures in addition to CABG or who planned to undergo emergency surgery. Furthermore, patients without vitD insufficiency or deficiency were also excluded from the study.

### Laboratory Tests

25-hydroxy (OH) vitD, calcium, and other biochemical and hematologic parameter levels were measured following a fasting period of 8 hours. Serum 25-(OH) vitD levels were measured by chemiluminescent immunoassay using a LIAISON analyser (DiaSorin Inc). VitD deficiency was defined as serum levels of 25-(OH) vitD <20 ng/ml. VitD insufficiency was defined as a level of 20-29 ng/ml. Plasma levels of 25-(OH) vitD >30 ng/ml were defined as normal. Oral vitD supplementation (50.000 U) was initiated 48 hours before CABG surgery.

### Statistical Analysis

Statistical analysis was performed using the SPSS (version 20.0, SPSS Inc., Chicago, IL, USA) software package. Continuous variables were expressed as mean ± standard deviation (mean± SD) and categorical variables were expressed as a percentage (%). The Kolmogorov-Smirnov test was used to evaluate variables distribution. Student's t-test was used to evaluate continuous variables showing normal distribution and Mann-Whitney U-test was used to evaluate variables that did not show normal distribution. A *P*-value <0.05 was considered statistically significant.

## RESULTS

The present study included 328 consecutive patients, of which 80 (24.3%) patients had vitD insufficiency and 56 (17%) had vitD deficiency. Table 1 presents the main characteristics of the patients with and without vitD supplementation. There was no significant difference between these two groups with regards to age (63.8±9.3 years *vs.* 62.7±8.9 years, *P*=0.761), female gender (46.3% *vs.* 48.1%, *P*=0.827), HT (62.5% *vs.* 63.7%, *P*=0.834), DM (37.9% *vs.* 35.4%, *P*=0.637), HL (32.8% *vs.* 34.7%, *P*=0.682), smoking history (24.6% *vs.* 27.1%, *P*=0.753), COPD (28.7% *vs.* 26.9%, *P*=0.756), left atrial diameter (38.9 mm *vs.* 39.3 mm, *P*=0.837), left ventricular EF (62.7±6.9% *vs.* 61.3±7.1%, *P*=0.843), and duration of hospitalization (8.1±2.3 days *vs.* 7.9±2.1 days, *P*=0.891) (Table 1).

**Table 1:** Patient's characteristics.

Patient's characteristics	Vitamin D supplementation		*P*
	(+) (n=68)	(-) (n=68)	
Age (years)	63.8±9.3	62.7±8.9	0.761
Female gender (%)	46.3	48.1	0.827
Hypertension (%)	62.5	63.7	0.834
Diabetes mellitus (%)	37.9	35.4	0.637
Hyperlipidemia (%)	32.8	34.7	0.682
Smoking history (%)	24.6	27.1	0.753
COPD, n (%)	28.7	26.9	0.756
Duration of hospitalization (days)	8.1±2.3	7.9±2.1	0.891
Left ventricular ejection fraction (%)	62.7±6.9	61.3±7.1	0.843
Left atrial diameter (mm)	38.9	39.3	0.837
Cross-clamp time (min)	74 (71.0-78.0)	75 (72-80)	0.672
Cardiopulmonary bypass time (min)	96 (84.0-101)	94 (80.0-98.0)	0.718
Beta-blocker therapy (%)	86	89	0.584
Statin therapy (%)	92	94	0.783
ACE-ı/ARB therapy (%)	72	70	0.867
Aspirin therapy (%)	96	93	0.567
Diuretics therapy (%)	23	21	0.738

ACE-ı/ARB=angiotensin-converting enzyme inhibitor/angiotensin receptor blocker; COPD=chronic obstructive pulmonary disease

Also, there was no significant difference between these two groups with regards to medications, such as beta-blockers (86% *vs.* 89%, *P*=0.584), statin (92% *vs.* 94%, *P*=0.783), ACE-ı/ARB (72% *vs.* 70%, *P*=0.867), aspirin (96% *vs.* 93%, *P*=0.567), and diuretics (23% *vs.*. 21%, *P*=0.738) (Table 1). And there was no significant difference between them with regards to cross-clamp time (74 min *vs.* 75 min, *P*=0.672) and cardiopulmonary bypass time (96 min *vs.* 94 min, *P*=0718) (Table 1).

In addition, there was no significant difference between these two groups with regards to biochemical and haematological parameters, such as hemoglobin (14.1±2.3 *vs.* 14.6±2.8 g/dl, *P*=0.652), platelet (293.5±46.9 *vs.* 289.3±49.6 10^3^/µL, *P*=0.563), white blood cell (7.9±3.7 *vs.* 8.2±3.4 10^3^/µL, *P*=0.857), creatinine (1.03±0.29 *vs.* 1.01±0.23 mg/dl, *P*=0.761), fasting plasma glucose (112.3±32.9 *vs.* 109.2±30.7 mg/dl, P=0.627), C-reactive protein (1.14±0.92 *vs.* 1.22±0.91 mg/dl, *P*=0.738), total cholesterol (186.3±43.1 *vs.* 181.3±44.9 mg/dl, *P*=0.613), high-density lipoprotein (37.1±9.7 *vs.* 39.3±11.5 mg/dl, *P*=0.514), low-density lipoprotein (163.4±43.8 vs 165.9±45.7 mg/dl, *P*=0.628), and triglyceride (183.1±56.7 *vs.* 178.1±54.7 mg/dl *P*=0.485) (Table 2).

**Table 2:** Laboratory parameters.

Laboratory parameters	Vitamin D supplementation		*P*
	(+) (n=68)	(-) (n=68)	
Hemoglobin (g/dl)	14.1±2.3 (14.7)	14.6±2.8 (14.9)	0.652
Platelet (10^3^/µL)	293.5±46.9 (302.8)	289.3±49.6 298.3)	0.563
White blood cell (10^3^/µL)	7.9±3.7 (8.7)	8.2±3.4 (8.9)	0.857
Creatinine (mg/dl)	1.03±0.29 (1.12)	1.01±0.23 (1.08)	0.761
Fasting plasma glucose (mg/dl)	112.3±32.9 (104.6)	109.2±30.7 (106.9)	0.627
C-reactive protein (mg/dl)	1.14±0.92 (0.97)	1.22±0.91 (0.99)	0.738
Total cholesterol (mg/dl)	186.3±43.1 (195.1)	181.3±44.9 (192.7)	0.613
High-density lipoprotein (mg/dl)	37.1±9.7 (39.3)	39.3±11.5 (41.4)	0.514
Low-density lipoprotein (mg/dl)	163.4±43.8 (184.2)	165.9±45.7 (182.7)	0.628
Triglyceride (mg/dl)	183.1±56.7 (192.6)	178.1±54.7 (189.4)	0.485
Vitamin D level (deficiency group) (ng/ml)	11.4±4.9	10.9±5.2	0.681
Vitamin D level (ınsufficiency group) (ng/ml)	24.6±3.7	24.9±3.9	0.837

Regarding to mean vitD level of insufficiency (24.6±3.7 *vs.* 24.9±3.9 ng/ml, *P*=0.837) and deficiency patients (11.4±4.9 *vs.* 10.9±5.2 ng/ml, *P*=0.681), there was no significant difference between treatment and control groups (Table 2).

Although the occurrence of POAF was not significantly different among treatment and control groups in patients with vitD insufficiency (31% *vs.* 33%, *P*=0.538), there was a significant difference between these two groups with regards to POAF in patients with vitD deficiency (18% *vs.* 29%, *P*=0.02) (Table 3).

**Table 3:** Prevalence of postoperative atrial fibrillation (POAF).

		Vitamin D supplementation		*P*
		(+) (n=68)	(-) (n=68)	
POAF (%)	Vitamin D insufficiency group, n (80)	31	33	0.538
	Vitamin D deficiency group, n (56)	18	29	0.02

## DISCUSSION

This study demonstrated that preoperative vitD supplementation was significantly preventive to the occurrence of POAF in patients with vitD deficiency while it was not preventive to the occurrence of POAF in those with vitD insufficiency. Beneficial effects of preoperative vitD supplementation might be associated with tend to more occurrence of POAF in patients with vitD deficiency.

AF is the most common sustained arrhythmia and it has a major impact on the morbidity and mortality of general population. The risk of AF increases up to 26% after the age of 40. It is estimated that AF may account for 10% to 15% of all strokes, with an associated mortality of up to 1.9-fold^[9]^. In previous studies, ageing, male gender, COPD, HF, DM, preoperative AF attacks, chronic renal disease, metabolic syndrome, increased cross-clamp times, increased inflammatory markers, and left atrial diameter were found to be independent variables predicting the development of POAF^[10]^.

Several potential mechanisms have described the association between vitD and AF^[11]^. VitD insufficiency was associated with endothelial dysfunction and subclinical atherosclerosis^[12]^. VitD regulates inflammatory responses and up-regulates the expression of anti-inflammatory cytokines, such as interleukin 10 (IL-10) and IL-6, according to in-vitro experiments^[13]^. Also, vitD regulates RAAS activity. Activated RAAS can lead to an increase in new-onset AF^[14]^. It is postulated that tissue RAAS can induce apoptosis of cardiomyocytes and can contribute to changes in atrial structure^[15]^.

There were inconsistent results regarding low vitD levels and AF. Demir et al.^[16]^ reported a strong relationship between vitD deficiency and non-valvular AF. Serum vitD level was significantly associated with AF in Chinese patients with persistent nonvalvular AF^[17]^. Hanafy et al.^[18]^ revealed the direct electromechanical effects on the left atrium after vitD administration and found that it could effectively prevent and terminate AF.

On the other hand, Rienstra et al.^[19]^ reported that vitD deficiency does not promote the development of AF. Additionally, Qayyum et al.^[20]^ also showed that there was no significant association between vitD deficiency and AF. Another prospective cohort study did not support the hypothesis that vitD level is associated with AF^[21]^.

In the same manner, there were inconsistent results regarding preoperative serum vitD level and POAF. POAF incidence was significantly higher in patients with vitD deficiency or insufficiency than in patients with normal vitD level in previous studies^[2,3]^. In our previous study, although there was a significant negative correlation between vitD and left atrial diameter, vitD level was not an independent predictor for POAF^[7]^.

Several studies demonstrated that platelets play a critical and precipitating role in AF occurrence as the volume and distribution width of platelets and the factors of platelet activity appeared to be significantly higher in AF patients than in sinus rhythm patients. Haematological indices red blood cell distribution width (RDW) and mean platelet volume (MPV) are currently recognised as risk factors for POAF. MPV, as a valuable haematological parameter in complete blood count test, could strongly predict the occurrence of paroxysmal, persistent, or permanent AF^[22-25]^. Our study did not include aforementioned haematological parameters such as MPV and RDW. In our study, there was no significant association between platelet count and POAF.

There are scarce data regarding the preventive role of preoperative vitD supplementation on POAF in patients with vitD insufficiency and deficiency. Therefore, we aimed to evaluate the preventive role of vitD supplementation. This study revealed that preoperative vitD supplementation was significantly preventive to the occurrence of POAF in patients with vitD deficiency while it was not preventive to the occurrence of POAF in those with vitD insufficiency. Preventive effect of vitD supplementation might be associated with direct electromechanical effects on the left atrium, regulating RAAS and inflammatory responses. Further randomised clinical studies with larger study population are needed in this field.

### Limitations of the Study

Our study has some limitations: first, AF was diagnosed by ECG monitoring only during hospitalisation process; second, this study has a small sample size; third, vitD level was measured at a single point in time; fourth, the lack of parathyroid hormone levels measurement; and fifth, the lack of haematological markers such as MPV and RDW.

## CONCLUSION

To the best of our knowledge, this study is the first to evaluate the preventive effect of vitD on POAF in patients with vitD insufficiency and deficiency. In this study, preoperative vitD supplementation was found to be significantly preventive to the occurrence of POAF in patients with vitD deficiency while it was not found to be preventive to the occurrence of POAF in those with vitD insufficiency. VitD deficiency should be considered in patients with POAF especially resistant to medical and electrical cardioversion. Further prospective and randomised studies with larger number of patients are required due to the relatively small number of patients in the present study.

**Table:** 

Authors' roles & responsibilities	
LC	Conception of the work; acquisition, analysis, and interpretation of data for the work, drafting the work; final approval of the version to be published
BÖ	Acquisition, analysis, and interpretation of data for the work, drafting the work; final approval of the version to be published
ZC	Conception of the work; acquisition, analysis, and interpretation of data for the work, drafting the work; final approval of the version to be published
HD	Acquisition, analysis, and interpretation of data for the work, drafting the work; final approval of the version to be published
